# Incidence of COVID-19 Vaccination-Related Uveitis and Effects of Booster Dose in a Tertiary Uveitis Referral Center

**DOI:** 10.3389/fmed.2022.925683

**Published:** 2022-06-22

**Authors:** Milton C. Chew, Shaan Wiryasaputra, Meihui Wu, Wei Boon Khor, Anita S. Y. Chan

**Affiliations:** ^1^Singapore National Eye Centre, Singapore, Singapore; ^2^Translational Ophthalmic Pathology Platform, Singapore Eye Research Institute, Singapore, Singapore; ^3^Ophthalmology and Visual Sciences Academic Clinical Program, Duke-NUS Graduate Medical School, Singapore, Singapore

**Keywords:** COVID-19, vaccination, uveitis, booster, coronavirus-19 disease

## Abstract

**Background:**

We report vaccine and booster-related uveitis in Singapore, a country with high vaccination and booster rates to highlight the differences and potential role of prophylactic treatment for sight-threatening infectious uveitis.

**Methods:**

Clinical data extracted from the de-identified uveitis database in Singapore National Eye Center. Six patients (eight eyes) developed uveitis within 14 days after undergoing COVID-19 vaccination (primary and/or booster).

**Results:**

All patients received two doses of COVID-19 vaccination, and 1.39% (6/431) developed COVID-19 vaccine-related uveitis. Fifty-percent% (3/6) with non-infectious anterior uveitis (NIAU) presented with a non-granulomatous anterior uveitis (AU). The remaining (3/6) presenting with a granulomatous AU were diagnosed with reactivation of cytomegalovirus, varicella-zoster virus and toxoplasma chorioretinitis, respectively. All the patients responded to definitive treatment specific to their diagnosis. The mean visual acuity at presentation was 0.36 ± 0.20 logMAR and improved to 0.75 ± 0.09 (*p* = 0.009). Mean time from vaccination to uveitis was 9.7 (range: 3–14) days. All patients developed uveitis after second vaccination dose. 16.67% (1/6) patients had a recurrence after the third booster dose. None of the three patients with infectious uveitis developed recurrence but had received maintenance therapy up to or during the booster.

**Conclusion:**

Uveitis after COVID-19 vaccination is uncommon. In our series, a higher rate of reactivations of latent infections was seen. With definitive treatment, all cases were self-limited without systemic sequelae. Prophylactic treatment during booster vaccine may prevent reactivation of sight-threatening infections and reduce morbidity although risk-benefits should be considered for individual patients given the low rate of occurrence.

## Introduction

The unprecedented nature of the Coronavirus (COVID-19) pandemic has led to the rapid development of COVID-19 vaccinations which are safe and effective (1). These vaccines can be broadly classified into mRNA-based vaccines (BNT162b2 Pfizer-BioNTech, and mRNA-1273 Moderna), inactivated whole virus vaccines (CoronaVac Sinovac, and BBIBP-CorV Sinopharm), viral vector vaccines (Ad26.COV2 Janssen Johnson & Johnson, and ChAdOx1 nCoV-19/AZD1222, Oxford-AstraZeneca), and protein subunit vaccines (NVXCoV2373, Novavax). Vaccine-associated uveitis is a known rare adverse event that has been previously described following other common vaccinations including those against Hepatitis A and B, human papillomavirus, Bacilli Calmette-Guerin (BCG), Measles-mumps-rubella, influenza, and varicella virus (2–5). COVID 19 vaccine-related uveitis has been associated with all vaccine sub types (6–11). A spectrum of disease presentation from mild anterior uveitis to severe sight threatening panuveitis has been reported (9–15). The Pfizer-BioNTech vaccine was most commonly associated with reported 57–63% of all COVID-19 vaccination-induced uveitis (7, 8).

With the emergence of the Delta and Omicron variants, worldwide concerns over the waning effects of the initial 2-dose COVID-19 BNT162b2 Pfizer-BioNTech vaccine has led to national vaccine booster campaigns advocating a third vaccination dose to boost humoral response (16, 17). Due to the scale of these COVID-19 national booster campaigns, patients who have experienced vaccine-associated events after their initial vaccinations are often anxious about booster-related flares. In Singapore, at the time of writing, 70% of our population of 5.45 million (18) have received a booster dose (19). The aim of this study is to report the incidence of COVID-19 vaccine and booster related uveitis in our center in comparison to published series and to discuss the role of prophylactic treatment for patients planned for repeat vaccination particularly in those with a prior episode of sight-threatening COVID 19 vaccine related uveitis.

## Methods

This was a retrospective cohort study in which clinical data was extracted from our Centre's uveitis database. From 1 January 2021 to 31 December 2021, patients who presented to the uveitis service at the Singapore National Eye Center with an acute uveitic episode within 14 days of receiving a COVID- 19 vaccination were reviewed. This study adhered to the tenets of the Declaration of Helsinki, and Institutional Review Board waiver was obtained due to the de-identification of the patient personal information.

The inclusion criteria included patients with *de-novo* uveitis (uveitis presentation for the first time), whilst patients with a history of uveitis were included only if they were in remission, defined as per the Standardization of uveitis nomenclature (SUN) inactive disease for at least 3 months after discontinuing all treatment for uveitis (20). Patients who had an active uveitis or were on treatment for uveitis at presentation were excluded. Data reviewed included patient demographics, medical, ophthalmic, and previous uveitis history, clinical presentation, and treatment outcome. The type of COVID-19 vaccine, including the time interval between each dose and booster, as well as the time interval between vaccination and onset of symptoms were also retrieved. In cases of *de-novo* uveitis, uveitis screening according to published uveitis diagnostic guidelines (21). This included a complete blood count, erythrocyte sedimentation rate (ESR), C-reactive Protein (CRP), Chest X-Ray, Venereal Disease Research Laboratory test, Treponema pallidum hemagglutination, Mantoux test, and QuantiFERON-TB Gold (QFT) was performed to exclude other causes. To exclude viral and toxoplasma infections in clinically suspicious cases, anterior chamber paracentesis was performed for polymerase chain reaction (PCR) detection of herpes simplex, varicella zoster, cytomegalovirus and toxoplasma DNA genome.

A *p*-value of <0.05 was considered to be statistically significant. The Statistical analysis with the Student's *t*-test was performed using SPSS for Windows version 25.0 (Released 2017. IBM SPSS Statistics for Windows. Armonk,NY: IBM Corp. USA).

## Results

From our database, a total of 431 patients had been seen in our uveitis clinic from 1 January to 31 December 2021. All 431 patients completed primary vaccination as per our country's National COVID-19 Vaccination program during this period. We identified six patients who met our inclusion criteria for COVID-19 related uveitis. Thus, we calculated the rate of COVID-19 related uveitis to be 6/431 over the 1 year period from 1 Jan to 31 December 2021 to be 1.39%. The majority of our patients received mRNA vaccines (Pfizer-BioNTech/ Moderna-Spikevax) with only 2/431 patients receiving non-mRNA vaccines (Sinovac-CoronaVac and BBIBP-CorV Sinopharm). No combination of different vaccines were noted. Patient demographics, vaccination related details, uveitis diagnosis, and clinical progress are summarized in Tables 1, 2. In brief, eight eyes of six patients (four Female, two Male), with a mean age of 50.0 years (range 28–71 years) were identified. Five (83.33%) patients had received the BNT162b2b Pfizer-BioNTech vaccine, whilst only 1/6 patient received the BBIBP-CorV Sinopharm vaccine. Two thirds (4/6) patients underwent a third booster dose 270 days after their second (Pfizer-BioNTech) dose. The mean time from vaccination to onset of uveitic flare was 9.7 days (range: 3–14 days). COVID-19 vaccine-related uveitis occurred after the second dose in 100% (6/6) patients. 83.33% (5/6) presented with blurring of vision, 50% (3/6) with red eye, and 16.7% (1/6) with eye pain. Half of the patients (3/6) were diagnosed with non-infectious anterior uveitis (NIAU) and the remainder (3/6) with infectious uveitis.

**Table 1:** Patient demographics, diagnosis and vaccine related findings.

**Patient number**	**Age**	**Gender**	**Eye**	**Immune status/** **other medical history**	**Uveitis history**	**Current presentation**	**Time interval between last uveitis attack and current uveitis (years)**	**Vaccine**	**Symptoms after which dose**	**Time interval between vaccination and symptoms (days)**	**Recurrence with booster**
1	64	Female	Right Eye	Immuno-competent Post cataract (2018)	Idiopathic anterior uveitis with CMO^π^ (Last attack in 2018)	Anterior uveitis with CMO	3	Pfizer	Second	14 days after second dose 3 days after booster	CMO recurred 3 days after booster and treatment regime repeated.
2	74	Male	Left Eye	Immuno-competent Hypertension Hyperlipidaemia	Nil	HLA-B27^†^ newly detected	First episode	Sinopharm	Second	3	No booster yet
3	31	Female	Left Eye	Immuno-competent	Nil	Nil	First episode	Pfizer	First and Second	10	No Booster Yet
4	71	Female	Left Eye	Immuno-competent Primary angle closure suspect s/p LPI^‡^	CMV^¥^-related Anterior uveitis. (Last attack in 2019)	CMV-related Anterior uveitis	2	Pfizer	Second	14	No recurrence with booster. On maintenance Ganciclovir 2%QDS^¤^ at booster
5	32	Female	Left Eye	Immuno-competent	Left eye toxoplasma chorioretinitis treated in 2016	Toxoplasma Chorioretinitis	5	Pfizer	Second	7	No recurrence with booster. Was given Bactrim prophylaxis.
6	28	Female	Right Eye	Immuno-competent	Nil	HZO-related anterior uveitis	First episode	Pfizer	Second	10	No recurrence with booster. Was on maintenance dose of oral Valtrex prior to booster

† *HLA-B27, Human Leukocyte Antigen B27*.

‡ *LPI, Laser Peripheral Iridotomy*.

π *CMO, Cystoid Macular Edema*.

¥ *CMV, Cytomegalovirus*.

*HZO, Herpes-zoster ophthalmicus*.

¤ *QDS, 4 times a day*.

**Table 2:** Patient clinical findings, treatment and outcome of COVID-19 associated uveitis.

**Patient number**	**T*y*pe of uveitis**	**Symptoms**	**Uveitis description**	**BCVA^†^ at presentation**	**BCVA at last follow-up**	**Intraocular pressure at presentation**	**Treatment given**	**Outcome and follow up after resolution**
1	Non-granulomatous Anterior Uveitis and CMO	BOV^‡^	Fine diffuse KPs ^π^, cells 1+, Retrolental cells 1+, CMO^¥^	6/12	6/6	15	G prednisolone acetate QDS G ketorolac TDS	Complete resolution; Quiescent 4 months without treatment
2	Non-granulomatous Anterior Uveitis	BOV, red eye	Diffuse fine KPs, cells +, Retrolental Cells occ	6/24	6/9.5	14	G prednisolone acetate Q3H, Occ dexamethasone ON	Complete resolution; Quiescent 4 months without treatment
3	Non-granulomatous Anterior Uveitis	Red eye	Fine diffuse KPs, cells 1+	6/7.5	6/6	15	G prednisolone acetate Q3H	Complete resolution; Quiescent 4 months without treatment
4	Granulomatous Hypertensive Anterior Uveitis	BOV	Mutton fat KPs, cells 2+	6/19	6/9.5	26	G ganciclovir Q2 G ketorolac TDS	Complete resolution; Quiescent 4 months without treatment
5	Reactivation of toxoplasma chorioretinitis	BOV, redness, floaters	Medium KPs, cells 2+, vitritis+, reactivation of old toxo scar	6/19	6/6	16	PO Sulfadiazine, Folinic acid, Pyrimethamine, Clindamycin	Complete resolution; Quiescent 1 month without treatment
6	Granulomatous Hypertensive Anterior Uveitis, concomitant HZO	BOV and pain	Mutton fat KPs, cells 1+, flare 1+	6/9	6/7.5	44	PO valacyclovir G prednisolone acetate QDS	Complete resolution; Quiescent 5 months without treatment

† *BCVA, Best-corrected Visual Acuity*.

‡ *BOV, Blurring of Vision*.

π *KP, Keratic Precipitate*.

¥ *CMO, Cystoid Macula Edema*.

*G, guttae; Occ, Ointment; PO, oral; Q2H, every 2 hourly; Q3H, every 3 hourly; QDS, 4 times a day; TDS, 3 times a day; ON, every night*.

The mean visual acuity (VA) at presentation (0.36 ± 0.20 logMAR) significantly improved with therapy (final mean VA, 0.75 ± 0.09; *p* = 0.009). Three patients (50%) had a known history of uveitis which were well-controlled and quiescent for a mean of 3.3 years (range: 2–5 years), while the remaining 3/6 patients presented with *de-novo* uveitis. None of the patients were on any topical or systemic treatment for uveitis prior vaccination.

### Non-infectious Uveitis (Recurrent Anterior and *de novo* Anterior Uveitis)

**Figure 1:**
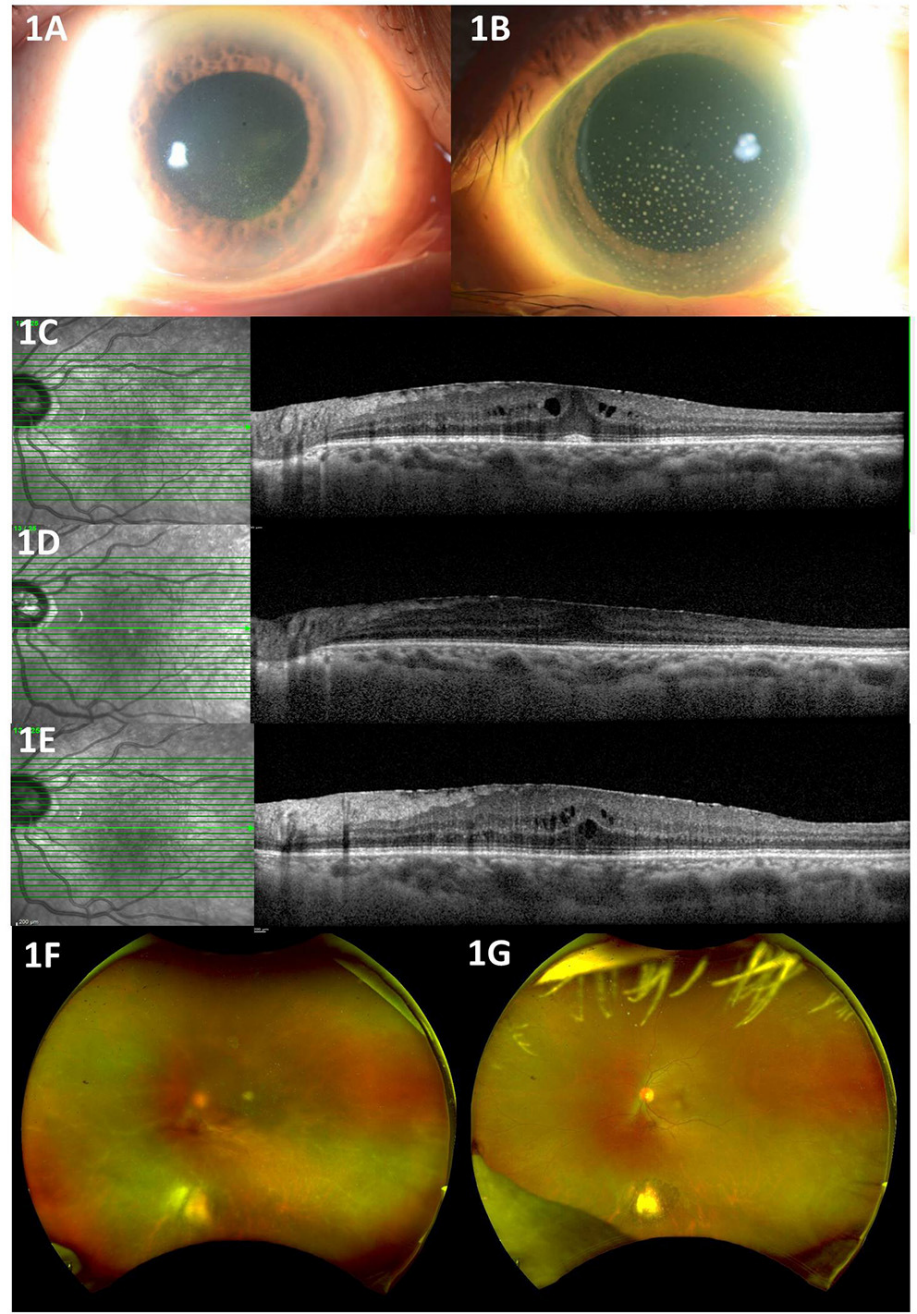
**(A)** Non-infectious anterior uveitis with fine diffuse keratic precipitates in Patient #2. **(B)** Granulomatous keratic precipitates in Patient #4. **(C)** Cystoid Macula Edema after COVID-19 vaccination in Patient #1. **(D)** Resolution of Cystoid Macula Edema with treatment in Patient #1. **(E)** Recurrence of Cystoid Macula Edema after COVID-19 booster in Patient #1. **(F)** Reactivation of toxoplasma chorioretinitis after COVID-19 vaccination in Patient #5. **(G)** Resolution of toxoplasma chorioretinitis with treatment in Patient #5.

Three patients (Patients #1, #2, #3) (Tables 1, 2) presented clinically with a normotensive, non-granulomatous anterior uveitis (AU). Patient #1 had a previous uveitis history whilst patients #2 and #3 had no past rheumatological or uveitis history. All patients presented with non-pigmented fine keratic precipitates with a predominantly inferior distribution (Figure 1A). We briefly describe the clinical history of two patients to highlight the recurrence of uveitis with both second and third booster doses, and a *de-novo* Human Leukocyte Antigen B27 (HLA-B27) AU associated with the Sinopharm^TM^ vaccination.

Patient #1 had a past history of anterior uveitis with cystoid macular edema (CMO) which had resolved in 2018 (Table 1). After the second dose, she developed recurrent AU with CMO in both eyes. She responded well with a course of topical corticosteroids of Prednisolone Acetate 1% that was tapered over 2 months (Figures 1C,D). As she was due for a booster injection 270 days after her second dose, the patient was given a standby dose of topical corticosteroids as she had expressed anxiety about a recurrence. Although she experienced a recurrence within 3 days of the booster dose characterized by redness, minor pain and blurring of vision, she did not use her standby medication but was reviewed earlier. She was found to have bilateral AU and CMO recurrence (Figure 1E) and successfully treated with topical steroids until resolution, and visual acuity recovery to baseline. After cessation of therapy, she had no recurrence of her uveitis or CMO over the next 4 months.

Patient #2 developed an acute AU 7 days after his second Sinopharm^TM^ vaccination dose. As he had no prior history of uveitis and this was his first presentation, work up was performed. He was found to be HLA-B27 positive, and the remaining uveitic workup was otherwise unremarkable other than a significantly raised ESR 79 mm/h but normal CRP 4.1 mg/L in the absence of systemic HLA-B27 related symptoms. He had no medical history of note to account for the elevated ESR. He responded well with a course of topical corticosteroids, and his ESR down-trended to 27 mm/h within a month. He has remained well with no recurrence for 3 months after cessation of therapy.

### Infectious Uveitis

Three patients (Patients #4, #5, #6; Tables 1, 2) presented with a reactivation of Cytomegalovirus (CMV), Toxoplasma chorioretinitis and Varicella-Zoster Virus (VZV), respectively (Tables 1, 2). Two patients had a previous history hypertensive CMV AU (Patient #4) and toxoplasma chorioretinitis (Patient #5) respectively, whilst the third presented with classical features of Herpes Zoster Ophthalmicus (HZO). The mean interval time of quiescence between the initial uveitis attack and the latest post-vaccination reactivation was 3.3 years (range: 2–5 years). All three patients presented with a granulomatous anterior uveitis (Figure 1B) after their second COVID-19 dose and underwent a third booster dose. We briefly describe each case highlighting their maintenance or prophylactic treatment at the time of booster. None of the three patients had a recurrence of infection after the booster.

Patient #4 had a previous history of left eye hypertensive CMV AU diagnosed by polymerase chain reaction (PCR) detection of aqueous CMV-DNA in 2019. Her CMV AU was quiescent and she was off medication for the past 2 years (Tables 1, 2). She presented with a similar left eye hypertensive granulomatous AU episode 14 days after undergoing COVID-19 vaccination in 2021. Topical Ganciclovir 2% therapy was restarted at 2 hourly (q2H) and she was on a maintenance dose of 4 times a day (QDS) during her booster dose.

Patient #5 had a history of previous left eye toxoplasma chorioretinitis and completed oral clindamycin and prednisolone treatment in 2016. She re-presented with a reactivation of the left eye latent toxoplasma infection 1 week after her second COVID-19 vaccination dose (Figure 1F). She recovered with toxoplasma systemic therapy (Tables 1, 2, Figure 1G). As she was anxious about a recurrence, she was given 2 weeks of trimethoprim-sulfamethoxazole 960 mg BD at the time of her booster. She did not have a recurrence of the inflammation after the booster.

Patient #6 had no previous uveitis. This was her first presentation of HZO 14 days after inoculation with COVID-19 vaccination. She presented with a typical trigeminal vesicular rash, sectoral scleritis and hypertensive anterior AU that persisted despite topical corticosteroids and oral acyclovir 800 mg 5 times a day for 4 days. Acyclovir was replaced with oral valacyclovir 1g three times a day (TDS) for 2 weeks, followed by 1g twice daily (BD) for a subsequent 2 weeks (Table 2). She had a mild persistent scleritis and AU for which she was kept on a maintenance dose of oral valacyclovir 500 mg every morning (OM) and tapering low dose topical steroids until complete resolution. All medication was stopped 5 days prior her booster without any recurrence of her uveitis.

## Discussion

Singapore has an aggressive vaccination strategy that has helped keep our healthcare service from being overwhelmed and kept death rates low despite the Delta and Omicron variants of SARS-CoV-2 increasing infection rates (22, 23). In our clinics, 100% of our (431) patients had received at least two doses of the nationally recognized vaccinations. The predominance of Pfizer vaccines reflects our local vaccination strategy where the government centers provided predominantly Pfizer-BioNTech vaccines with only limited centers providing Moderna-Spikevax (19). Sinovac-Coronavac and Sinopharm^TM^ were officially available only in the latter part of 2021 (24), as such only 2/ 431 patients reported the use of this vaccine. In our clinic, we report a low (6/431, 1.39%) rate of COVID-19 related vaccination uveitis. None of our patients developed systemic diseases or sequalae. Although patient #2 carried the HLA-B27 gene, he did not have systemic involvement clinically nor radiologically. Similarly, studies in patients from rheumatological clinics also indicate a low rate (4.4%) of flares secondary to COVID-19 vaccines (25).

In our study, we used strict inclusion criteria to minimize the possibility of coincidental recurrences and better establish causality. Our patients were quiescent based on the SUN criteria (20) for a mean of 3.3 years, and were not on any topical or systemic treatment at the time of vaccination. Using the Naranjo Algorithm- adverse drug reaction probability scale (6, 26), a probability score of +6 was achieved in the two patients who experienced repeat flares after two separate vaccine doses (Patient #1 and #3) which suggests a probable association. Our remaining patients scored only +4 suggesting a possible association with COVID-19 vaccination. Using the WHO-UMC for standardized case causality assessment in adverse drug reactions (27), our patients with a +6 Naranjo scale could be classified as “certain” whilst the those with +4 Naranjo scale would be classified as “likely/ possible.” The close temporal relationship between vaccination to presentation further favors a possible correlation rather than a coincidental occurrence.

Our low rates of COVID-19 vaccination-related uveitis (6/431, 1.39%) also corroborates with rheumatologic series that report a low rate of rheumalogical flares (4.4%) in patients with autoimmune and musculoskeletal disease (25). We emphasize the importance of a strict inclusion criteria as the inclusion of coincidental uveitis may result in over-reporting the occurrence of COVID-19 vaccine related uveitis which could adversely affect the uptake of booster doses especially during this Omicron variant phase.

This study also highlights a higher proportion of infectious uveitis, in comparison to the reports from multinational groups (7), Israel (6), and the Middle East (11) where a higher predominance of NIAU was reported. We also did not report significant posterior uveitis other than toxoplasma retinochoroiditis although retinal involvement has been described (7, 10, 11, 14).

As with any uveitis, the management of COVID-19 vaccine-related uveitis is a diagnosis of exclusion, and thus the identification of an underlying etiology with the exclusion of infections remains important. Apart from the standard uveitis questionnaires for previous uveitis, medical history and constitutional health symptoms, during the COVID-19 pandemic, vaccination status and past COVID-19 infection history are additional questions that clinicians should enquire about. As systemic uveitis screen are routinely performed in severe or bilateral uveitis and/or in elderly individuals for the exclusion of infective or autoimmune causes (20), in patients with recent COVID-19 vaccine or infections, it is also important to know the effects of these on the biochemical results such as ESR and CRP (28). Previous studies suggest COVID-19 vaccine and infections can elevate these inflammatory markers. Our patient #2 had an elevated ESR of 79 mm/h, in the absence of clinico-radiological evidence of HLA-B27 spondylopathy nor systemic symptoms, that may reflect the recent COVID-19 vaccine immune response. As his CRP was within normal limits, it also suggests that the acute inflammation from the HLA-B27 AU may play a lesser role. Thus, it may be possible to postulate that his recent vaccine may have triggered an underlying immune dysregulation in a genetically susceptible individual to result in this first presentation of uveitis. This is supported by reports that the COVID-19 vaccines may to trigger a *de-novo* autoimmune phenomena (29).

Appropriate definitive treatment is essential for the fast resolution of uveitis symptoms and visual recovery. Similar to the other reports on COVID-19-related uveitis, our study also found that the AU was self-limited in nature, responsive to corticosteroids, and did not result in systemic sequelae. Our patients with NIAU recovered with only topical corticosteroids therapy. Patient #2's ESR also returned to normal within a month. Of interest, Patient #2 received a non-mRNA, Sinopharm ^TM^ vaccine suggesting that inactivated viral-based vaccines may also incite a significant immune response.

Keratic precipitates (KPs) are useful clues for differentiating between granulomatous and non-granulomatous inflammation. In our series, NIAU presented with fine, non-pigmented KPs (Figure 1A), whilst the infectious AU from reactivation of VZV, CMV, toxoplasmosis uveitis, presented with granulomatous KPs (Figure 1B). For the non-uveitis trained ophthalmologist, the presence of granulomatous KPs should alert the individual to exclude such infections and not presume that all vaccine-related uveitis is immune-mediated. The type of infection that may reactivate may be influenced by geography with infections common in Asia such as toxoplasma and CMV being less common in the West. Given the epidemiological differences of viral-induced uveitis in Asia compared to the West (30), it is important to be aware of this. In our study, there was 50% infection and 50% NIAU. Although our series is small, there is a trend to suggest that infectious uveitis may also contribute to COVID-19 related uveitis in addition to NIAU, and we highlight this to increase awareness of possible COVID-19- vaccine related reactivation of infectious uveitis. Furthermore, VZV reactivation not limited to ocular involvement has also been associated with COVID-19 vaccinations (31–34).

The underlying pathophysiology for vaccine-related uveitis is largely unknown, but has been postulated to be an autoimmunity triggered by the vaccines (29, 35). It can be possibly due to a combination of various mechanisms including molecular mimicry, production of particular autoantibodies, antigen-antibody hypersensitivity reactions, as well as the role of certain vaccine adjuvants (3, 36, 37). Vaccination triggers a cascade of pro-inflammatory Type 1 Interferon expression, which results in the protective immune response, but can also trigger the production of autoantibodies responsible for an autoimmune phenomena (37). Specifically for mRNA vaccines, mRNA can bind to Toll-like receptors (TLR) as a form of molecular mimicry and activate several pro-inflammatory cascades including that of the Type 1 interferon response (38). Other studies suggest that it is the use of TLR7/8 and TLR9 agonists as adjuvant generates and amplifies the autoreactive immune responses (29). Age-associated B cells (ABCs), which are known to be associated with autoimmunity, expand gradually with age in healthy individuals but proliferate robustly in response to TLR7 or TLR9 stimulation, and may thus result in the production of autoreactive immunoglobulins, leading to autoimmune disease flare or new onset disease (39, 40). These ABCs may also expand in infectious diseases such as COVID-19 to contribute to the autoimmune phenomena (29).

Although COVID-19 related uveitis has been reported after the first vaccination dose (7), 5/6 of our patients presented after the second dose of vaccine. Two patients had more than on COVID-19 related uveitis events (Patient #3 after the second dose; and Patient #1 after the third booster dose). The precise mechanisms for higher reactogenicity after the second or subsequent doses of Covid-19 mRNA vaccine are still unclear, but several studies have shown that systemic adverse reactions after the second dose are associated with higher levels of anti-spike IgG protein in the serum post-vaccination (41–44). This higher levels of anti-spike IgG protein has been reported to correlate with increased naïve and transitional B cells and activated CD8+ T cells (45). Although the serological data for these patients is unavailable, it is likely that the first dose served to prime the immune system and the cumulative effects resulted in a shift in the B cell and CD8+ T cell population that may have triggered the autoimmune flare or reactivation of the latent infections (46). For patient #3 who experienced a flare after the first dose, the individual variation in immune responses to the vaccine, may have triggered a significantly higher anti-spike IgG protein and thus a proportionately higher number of naïve and transitional B cells and functional spike-specific CD8+ T cells. This has been reported in some vaccine recipients as early as 1 week after the first vaccine dose (47, 48).

For a latent viral reactivation (specifically for VZV and CMV), it has been postulated to be related to the robust T-cell response following vaccination (36) which causes a massive shift and increased CD8+ T cell and T-helper type 1 (Th1) CD4+ T cells specific for the spike protein or other antigens of SARS-CoV-2. This can lead to a paradox of VZV or CMV-specific CD8+ cells temporarily unable to control the latent virus, and hence precipitating a reactivation (31). Similarly in Toxoplasma infection, the immune response is largely Th1 driven, which the parasite-specific CD4+ and CD8+ T cells are responsible for cell-mediated immunity to the pathogen (49, 50). Likewise, the massive shift of T-cell shift following vaccination can similarly precipitate a toxoplasma reactivation.

The role of prophylactic treatment prior to future covid-19 vaccination booster remains undetermined. Studies have shown reduced risk of recurrence in ocular toxoplasmosis (51, 52) and viral uveitis (53, 54), to prevent a recurrent flare, prevent its associated complications, and reduce treatment burden. In our series, patient #5 with ocular toxoplasmosis expressed anxiety at the potential recurrence of visual loss when it was time for the booster and requested prophylactic therapy. This may reflect our patients' concerns and although the role of prophylactic treatment to prevent flares from COVID-19 vaccination may not be warranted for all patients given the low incidence of flares from our series, it may have a role in alleviating the stress addressing vaccine and booster hesitancy. We prescribed trimethoprim-sulfamethoxazole as prophylaxis and she did not experience a recurrence with the booster vaccination. Patients #4 was on maintenance dose of topical ganciclovir 2% QDS at the time of her booster and did not report any uveitic flares. Patient #5 had stopped her valacyclovir 500 mg OM 5 days prior her booster which was also uneventful.

In contrast, patient #1 with NIAU and CMO, who was not on any treatment at the time of booster, developed a flare within 3 days of her booster dose. She responded with only topical therapy which suggests that for immune-mediated uveitis, the higher degree of reactogenicity with subsequent booster dose can be dampened with a short course of prophylactic corticosteroids, and prophylactic treatment is probably not required, although advice for a prompt review on potential recurrences should be given.

Although others have reported on COVID-19 related uveitis and in larger series, the strength of our study is that we determined the incidence of COVID-19-related uveitis in a highly vaccinated population. We also report data on the potential flares secondary to the third booster dose which is becoming increasingly indicated. In our series, our patients presented with self-limited NIAU or infections endemic to our region. We also want to highlight the importance of the appearance of a granulomatous KPs, which although not definitive for diagnosis, should raise suspicions to evaluate for a reactivation of a latent infection. We also demonstrated that COVID-19 induced vaccination can occur in independent of the type of vaccine, although the type of vaccine our patients received largely reflected our government's vaccination strategies where mRNA vaccines (BNT162b2 Pfizer-BioNTech predominantly) was the vaccine of choice, and the BBIBP-CorV Sinopharm only available later. This is also one of the limitations of our studies as we were unable to evaluate the risk from other vaccines. However, since 2-dose vaccination status in our cohort was 100%, we are able to demonstrate that the rate of COVID-19 vaccine uveitis was low. The other limitations of our study included the retrospective design of the study, as well as the small number of patients in our cohort. In addition, as a tertiary referral center for uveitis, we may see cases that are more complex which may have skewed the ratio of NIAU to infective causes.

## Conclusion

In summary, we present a series demonstrating the clinical spectrum of COVID-19 vaccination-induced uveitis. From our study and others, COVID-19-vaccination induced uveitis is rare, with the majority being self-limiting, with good responses to treatment and no long-term sequelae. However, we highlight that in our region of Southeast Asia where viral and toxoplasma infections may be more common, reactivation of such infections may occur at a higher proportion. The presence of granulomatous KPs may be a useful sign to alert the clinicians in the absence of clinical history or other classical signs. Early recognition and definitive therapy for COVID-19 vaccination-induced uveitis remains the mainstay for excellent clinical outcomes. Although some of our patients were on prophylactic or maintenance doses for infectious uveitis during their booster, we suggest a balance of risk vs. benefits discussion with individual patients and close monitoring in view of the low rates of recurrence in our series. As there is increasing need for booster vaccination with the recent emergence of the Omicron; it is important for ophthalmologists and generalists to be cognizant of COVID-19 related uveitis and be ready to advise patients who may want to defer vaccination or booster injections for fear of uveitis flares as the benefits of COVID-19 vaccination and booster still outweigh the risk of uveitis.

## Data Availability Statement

All data generated or analyzed during this study is included in this published article. The datasets used and/or analyzed during the current study are also available from the corresponding author on reasonable request.

## Ethics Statement

This study was performed after being granted a waiver of ethical review for the study on human participants by our local institutional review board in accordance with national legislation and institutional requirements as anonymised data sets were used. Written informed consent for participation was not required for this study in accordance with the waiver of national legislation and the institutional requirements.

## Author Contributions

MC and AC contributed to conception, design of the study, and wrote the first draft of the manuscript. MC performed the statistical analysis. WS, WM, and KB wrote sections of the manuscript. All authors contributed to manuscript revision, read, and approved the submitted version.

## Funding

This paper was funded by our institutional SingHealth Foundation (SHF)- Singapore National Eye Centre (SNEC) Fund #0620-5.

## Conflict of Interest

The authors declare that the research was conducted in the absence of any commercial or financial relationships that could be construed as a potential conflict of interest.

## Publisher's Note

All claims expressed in this article are solely those of the authors and do not necessarily represent those of their affiliated organizations, or those of the publisher, the editors and the reviewers. Any product that may be evaluated in this article, or claim that may be made by its manufacturer, is not guaranteed or endorsed by the publisher.
